# Objective assessment of spasticity by pendulum test: a systematic review on methods of implementation and outcome measures

**DOI:** 10.1186/s12938-020-00826-8

**Published:** 2020-11-09

**Authors:** Fariborz Rahimi, Reza Eyvazpour, Nazila Salahshour, Mahmood Reza Azghani

**Affiliations:** 1 Department of Electrical Engineering, Faculty of Engineering, University of Bonab, 5551761167 Bonab, East Azerbaijan Iran; 2 Department of Electronics, Faculty of Electrical and Computer Engineering, University of Tabriz, Tabriz, Iran; 3 Department of Biomechanical Engineering, Faculty of Biomedical Engineering, Sahand University of Technology, Tabriz, Iran

**Keywords:** Spasticity, Wartenberg pendulum test, Objective assessment, Outcome measure

## Abstract

**Background:**

Instrumented pendulum test is an objective and repeatable biomechanical method of assessment for spasticity. However, multitude of sensor technologies and plenty of suggested outcome measures, confuse those interested in implementing this method in practice. Lack of a standard agreement on the definition of experimental setup and outcome measures adds to this ambiguity and causes the results of one study not to be directly attainable by a group that uses a different setup. In this systematic review of studies, we aim to reduce the confusion by providing pros and cons of the available choices, and also by standardizing the definitions.

**Methods:**

A literature search was conducted for the period of 1950 to the end of 2019 on PubMed, Science Direct, Google Scholar and IEEE explore; with keywords of “pendulum test” and “Spasticity”.

**Results:**

Twenty-eight studies with instrumented pendulum test for assessment of spasticity met the inclusion criteria. All the suggested methods of implementation were compared and advantages and disadvantages were provided for each sensor technology. An exhaustive list categorized outcome measures in three groups of angle-based, angular velocity-based, and angular acceleration-based measures with all different names and definitions.

**Conclusions:**

With the aim of providing standardized methodology with replicable and comparable results, sources of dissimilarity and ambiguity among research strategies were found and explained with the help of graphical representation of pendulum movement stages and corresponding parameters on the angular waveforms. We hope using the provided tables simplify the choices when implementing pendulum test for spasticity evaluation, improve the consistency when reporting the results, and disambiguate inconsistency in the literature.

## Background

Spasticity according to European Thematic Network to Develop Standardized Measures of Spasticity is ‘‘disordered sensory-motor control, resulting from an upper motor neuron lesion, presenting as intermittent or sustained involuntary activation of muscles’’ [1]. This complex motor disorder which is caused by diverse sources of upper motor neuron lesions, affects patients with stroke [2], spinal cord injury [3], traumatic brain injury [4], multiple sclerosis [5] and cerebral palsy [6]. Spasticity can create severe pain and hence may negatively affect the quality of life. Spasticity can interfere with movement and can lead to stiff, painful joints [7, 8]. This disorder affects more than 12 million persons in the world and interferes with the natural movements of the patients [9, 10]. Billions of dollars are spent in pharmaceutical industry to develop anti-spasticity drugs, though lack of repeatable and objective outcome measures hinders the success [8]. It is generally agreed that spasticity is easy to recognize, but not so easy to quantify. Quantitative assessment of spasticity and spasms is crucial in evaluating treatment interventions. Although quantitative clinical scales have been proposed and are currently in use [11–14], but they lack objectivity. This means that with lower resolution and repeatability, which are inherent with subjective measures, perceived level of spasticity or its improvement/worsening can contain errors or small changes be unrecognized.

To overcome objectivity issue, one of the promising methods that was introduced about 70 years ago was pendulum test [15]. However, such objective quantification systems that can be easily used in clinics are still missing; although care should be taken that processing techniques may also affect numerical outcomes of any test [16]. Many attempts can be found in the literature that aimed at providing instrumented pendulum test of spasticity [17–19] and at times for stiffness [20]. Still, when it comes to methods of implementation and choice of outcome measures, the literature is very diverse and at times non-consistent. In other words, with each group of researchers having their own methodology, although the level of spasticity can be relatively assessed in each experiment data, comparing the results between experiments are very difficult. This makes practical use of the published research difficult, particularly for those who want to actually implement this method. Although Review studies can be found on pendulum test of spasticity, they have mainly focused on psychometric properties of this method [21, 22]. Although these reports state that pendulum test method is reliable and valid, they have not focused on the compatibility of results between researchers with different methodologies. In this article we have exhaustively searched the published scientific literature to come up with a clear list of options for practical methods of implementation. We have further tried to find the sources of incompatibility in the reported results and particularly considered effect of available choices of outcome measures for pendulum test of spasticity.

## Results

### Description of studies

**Fig. 1:**
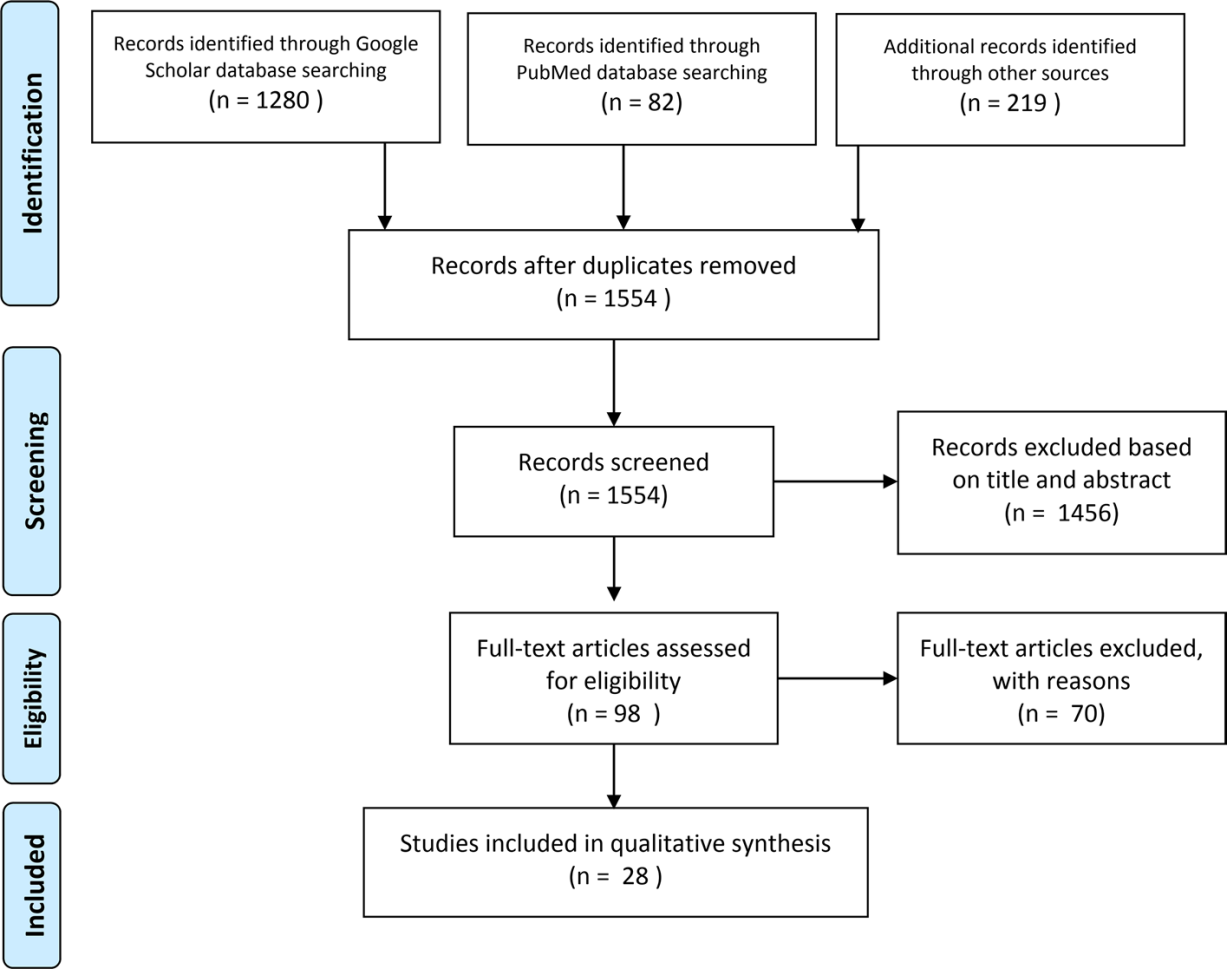
Flow chart of the search and selection process of the systematic review of objective assessment of spasticity by pendulum test for methods of implementation and outcome measures

As summarized in Fig. 1, initial search provided 1581 articles. After removing duplicates, 1554 articles remained from which 1456 were excluded because of not being eligible by examining the title and abstract. From the remaining 98 articles, 70 were removed based on full text assessment for eligibility. Three more relevant studies were found through the articles’ references. Therefore, 28 articles met the eligibility criteria and were included in the review (Table 3).

### Assessment of spasticity

Methods of assessment of spasticity can be broadly categorized into clinical, biomechanical, and electrophysiological [23] methods. Clinical methods, that are gold standards in clinic routines, are semi-qualitative. Although clinical scales have been proposed to provide quantitative outcomes, they suffer from lack of objectivity. This means, there is usually inter- and intra-clinician differences while evaluating levels of spasticity; and the resolution is often lower than when using a fully objective device. Biomechanical and electrophysiological methods involve sensors or instruments and can provide objective outcome measures.

A handful of clinical measures such as (CSI: composite spasticity index [24], HAT: hypertonia assessment tool [25], and TSS: triple spasticity scale [26], etc.) have been proposed and used in clinical assessments. However, the most routine method of assessment of spasticity by clinicians are the following four scales that are compared in detail in Table 1, Table 2: Ashworth Scale (AS) [11], Modified Ashworth Scale (MAS) [12], Tardieu Scale (TS) [13], and Modified Tardieu Scale (MTS) [14].

**Table 1:** Comparison of Ashworth and Modified Ashworth scales

Score	AS [11]	Changed in [12]
0	No increase in tone	No change
1	Slight increase in tone manifested by a “catch” when the limb is moved in flexion/extension	Slight increase in tone manifested by a catch, release or minimal resistance at the end of range of motion(ROM) when the limb is moved in flexion/extension
1 +		Slight increase in tone manifested by a catch, followed by minimal resistance throughout the remainder(less than half)of ROM
2	More marked increase in tone, but the limb is easily moved through its full ROM	More marked increased in tone through most of the ROM, but limb is easily moved
3	Considerable increase in tone- passive movement difficult	No change
4	Limb rigid in flexion and extension	No change

**Table 2:** Comparison of Tardieu and Modified Tardieu scales

Score	TS [13]	Changed in: MTS [14]
0	No resistance throughout the course of the passive movement	No change
1	Slight resistance throughout the course of passive movement	No change
2	Clear catch at precise angle, interrupting the passive movement, following by release	No change
3	Unsustained clonus(less than 10 s when maintaing the pressure) occurring at a precise angle, followed by release	No change
4	sustained clonus(more than 10 s when maintain the pressure) occurring at a precise angle, followed by release	No change
5	Joint is immovable	No change

All these scales assess spasticity by manual estimation of increased resistance of specific muscle groups to passive motion. From historical point of view, in 1954 Tardieu et al. introduced a 6-point scale to assess spasticity for the first time which was speed dependent (explained in the following paragraphs).

A decade after that and independently, in 1964, Ashworth proposed a 5-point scale which did not take the velocity dependence of spasticity into account. This scale was modified in 1987 by Bohannon and Smith to deal with the accumulation of most of the scores towards the lower end of the scale. They performed this by including an extra category (1+ , Table 1), making it a 6-point scale, and also modified the definitions slightly. In 1999, Boyd and Graham tried to standardize procedure of spasticity assessment and provided the modified TS (kept the 6-point system). MTS quantifies two angles: angle of ‘catch’ (stretch reflex threshold, measured at high speed) and the full range of motion (ROM, an angle measured at low speed). The difference between the two angles is suggested to be an indication of dynamic spasticity (dynamic tone).

Table 1 compares clinical evaluation and scores of spasticity by Ashworth and modified Ashworth scales. Table 2 demonstrates the method of clinical evaluation of spasticity by both Tardieu and modified Tardieu scales. The differences are only in performing the test and interpretation of the results. For passive stretch in MTS only two speeds are utilized. At slow speed, passive range of motion is found as an angle; and at fast speed, spastic reaction is evaluated as another angle. Tardieu et al. in 1954 experimented on passive moment of elbow at different speeds and had concluded that a stretch reflex was elicited at a specific speed (and above that) for each respective patient. The difference between these two angles is the true measure of spasticity for each patient.

Although clear protocols are used to teach and implement clinical scales, when a clinician repeats a scale or two clinicians implement the same scale on assessing a person’s level of spasticity, the results may not be the exact same. Objective and quantitative assessment methods make evaluation independent of individual judgement and often increase the sensitivity. They resolve the issues of inter- and at-rater variability as well. From objective and quantitative methods of spasticity assessment, in this review, we focus on one of the most popular biomechanical methods which is pendulum test.

### Pendulum test

**Fig. 2:**
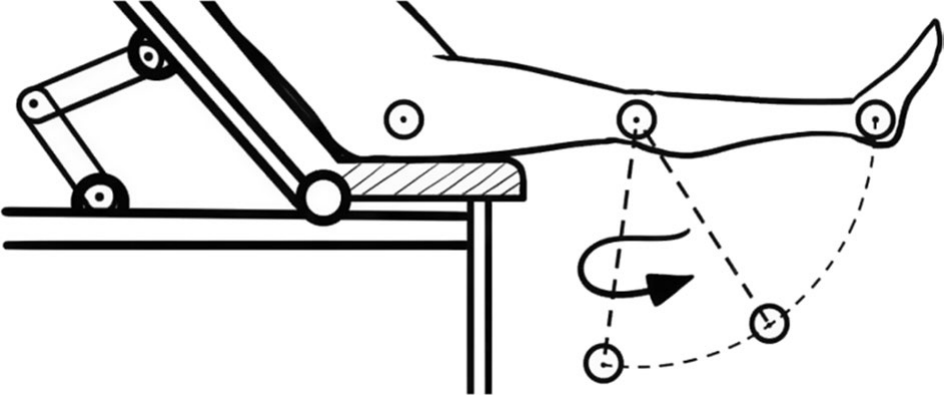
Experimental setup for pendulum test of spasticity at the knee level

Introduced in 1951 Wartenberg’s pendulum test was designed to objectively assess knee spasticity in a free oscillation when the leg is first raised to horizontal position and then dropped [15]. This test is subjective, simple and quick to implement, reproducible, non-invasive, and is non-intimidating to the children or individuals with cognitive impairments. However, it has its own shortcomings. For example, the results are completely affected by the level of relaxation and by the form of sitting. This test was initially aimed explicitly at the lower limb, but modified versions were later used for upper extremities as well. Boczko et al. [27] modified it in 1958 as an instrumented test with a flashlight and camera to capture the trace of oscillation. Later, many sensor technologies have been used in instrumented pendulum test (Table 4). The common procedure to perform this test starts with the participant sitting in a relaxed position and the leg to test hanging freely over the edge of a seat or a table (Fig. 2). Then the examiner lifts the leg to a horizontal position and lets it swing freely. From this free oscillation, many measures can be extracted to quantify the level of spasticity. Since the test accuracy depends completely on the relaxation of the leg being tested (no voluntary muscle contraction), methods of confirmation of relaxation have been proposed. The two common methods are using Electromyography (EMG) [6, 9], and using phase plane diagram of angle versus angular velocity [28]. Numerous studies implemented this biomechanical test in diverse groups of patients including normal and spastic adults [10], healthy people and those in vegetative state [18], and on children with cerebral palsy [29]. All the results showed a good test–retest and inter-rater reliability of measurement, and that any of the used sensors can be reliably used to characterize leg kinematics during the Wartenberg pendulum test and provide quantitative evaluation of spasticity.

To the best of our knowledge, there is no a comprehensive review that compares hardware or technologies for this test and also lists all possible outcome measures. So in this review, we have tried to provide a complete list of all possible and clinic-friendly technologies for implementation of this test along with their main advantages and drawbacks. We have also tried to come up with a comprehensive list of outcome measures.

### Methods of implementation of pendulum test

Since its introduction in 1951, the pendulum test was implemented using various devices or technologies, and in different groups of patients with spasticity. However, it took 7 years until the first objective data collection was performed by Boczko and Mumenthaler using a battery-fed light and a video camera [30]. Most of the studies (93%) have focused on the spasticity at the knee joint, and a few tried to examine spasticity with pendulum test at elbow or ankle joints. In more than half a century, researchers have used this test in objective assessment of spasticity in many diseases including Stroke, Multiple Sclerosis (MS), Cerebral Palsy (CP), Spinal Cord Injury (SCI), lesions (cerebral or in central nervous system), paraplegia and hemiplegia, and trauma. Some of the studies (54%) compared healthy participants with their patient population. Almost half of these studies also used clinical scales either to choose the range of spasticity in their patient population [17, 31] or to compare their outcome measures according to the divisions that clinical scales provided [6, 18]. Table 3 presents available studies that used pendulum test in chronological order, indicating the joint under study, participant groups, main technology for implementation of the test, and whether clinical scales are used in the study. There have been important theoretical studies such that Jikuya et al. [32] that have modeled spasticity during the pendulum test, or Kusuhara et al. [30] and Yamamoto et al. [33] that have theoretically examined advantages of using two linear accelerometers in pendulum test, but not included in the table because of no experimental data on patient population.

**Table 3:** Summary of the studies and technological implementation of Wartenberg’s pendulum test

Author/year	joint	People under study (number of participants)	Main technology	Compared to clinical scale
Couvée, 1968 [52]	Knee	Paraplegia (*N* = 6) Healthy (*N* = 5)	Potentiometer	NC
Bajd, 1984 [38]	Knee	SCI (*N* = 10) Hemiplegics (*N* = 5)	Electro goniometer Tachometer	NC
Leslie, 1992 [42]	Knee	MS (*N* = 14)	Electro goniometer	AS
Stillman, 1995 [19]	Knee	Healthy young, middle aged, elderly (*N* = 77)	Video camera	NC
He, 1997 [44]	Knee	MS (*N* = 46)	Electro goniometer	NC
Kaeser, 1998 [34]	Knee	Healthy (*N* = 40) Spastic (*N* = 9)	Electro goniometer Ultrasound	AS
Greenan Fowler, 2000 [6]	Knee	CP (*N* = 30) Healthy (*N* = 10)	Electro goniometer	MAS
Cavorzin, 2001 [53]	Knee	Spastic (*N* = 15) Healthy (*N* = 10)	Potentiometer EMG	AS
Nordmark, 2002 [17]	Knee	CP (SDR) (*N* = 20)	Electro goniometer EMG	MAS
Lin, 2003 [47]	Elbow	Stroke (*N* = 11) Healthy (*N* = 11)	Electro goniometer EMG	AS
Syczewska,2009 [29]	Knee	CP (*N* = 21) Trauma (*N* = 6), DS (*N* = 3)	Vicon Motion Capture ENG	NC
Bohannon, 2009 [46]	Knee	Chronic stroke (*N* = 8)	Polhemus Liberty magnetic position tracking system	AS
Sterpi, 2013 [18]	Knee	Severe cerebral lesion (*N* = 11) healthy (*N* = 10)	Inertial sensor (Accelerometer, Gyroscope, Magnetometer)	MAS
Tancredo, 2013 [31]	Knee	SCI (*N* = 11)	Accelerometer, Electro goniometer	MAS
Lemoyne, 2013 [54]	Ankle	Healthy (*N* = 1)	Accelerometer (IPhone wireless)	NC
Azevedo, 2013 [55]	Knee	SCI (*N* = 5) (5 Time)	Electro goniometer	NC
Szopa, 2014 [43]	Knee	CP (*N* = 36) Healthy (*N* = 18)	Accelerometer	DAROM
Yeh, 2016 [45]	Knee	Stroke (*N* = 13) Healthy (*N* = 3)	Electro goniometer (Two) Gyroscope (Wii remote)	MAS
Vargas Luna, 2016 [41]	Knee	SCI (*N* = 4)	Goniometer Video tracking	AIS
Bui, 2017 [40]	Knee	ARSACS (*N* = 13) Healthy (*N* = 32)	Gyroscope Accelerometer	MAS
Popovic-Maneski, 2017 [37]	Knee	Chronic SCI (*N* = 2) Healthy (*N* = 5)	Accelerometers (two) Gyroscope Encoder (Hall-effect) EMG	MAS
Popovic-Maneski, 2018 [39]	Knee	SCI (*N* = 9) Healthy (*N* = 6)	Absolute joint angle encoder Gyroscope EMG Electrode	AS
Aleksić, 2018 [39]	Knee	SCI (*N* = 1) Healthy (*N* = 1)	Marker-based system Joint angle encoder and IMU	NC
Popović, 2018 [56]	Knee	CNS lesion (*N* = 0)	Accelerometers (two) Gyroscope Encoder (Hall-effect) EMG	AS
Whelan, 2018 [36]	Knee	ABI (*N* = 45) MS (*N* = 14) CP (*N* = 12) SCI (*N* = 22)	Fiber-optic goniometer (FOG) EMG electrode	MAS

## Discussion

### Technologies used in objective pendulum test

Since the method of assessment of spasticity through pendulum test, and the processing steps to reach to the outcome measures are fully dependent on the implemented technology, this aspect is separately assessed in the literature. As Table 3 shows, variety of technologies, from ultrasound sensors [34] to camera-based [29, 35] or fiber-optic goniometers [36], have been used in the devices that implemented the test. However, these technologies differ in terms of price, stability, repeatability, ease of setup, complexity of signal post-processing, etc. advantages and disadvantages are compared in Table 4.

**Table 4:** Summary of the popular technologies used in objective pendulum test, and their advantages and shortcomings

Technology	Advantage	Disadvantage
Potentiometer (angle)	Simple, low cost and suitable for online computer analysis [38]	Higher errors; lower stability; difficult to attach & hindering knee-joint motion [30]
		Need for differentiation to get angular velocity / acceleration [57]
Goniometer (angle)	Easy to attach [57]	Questionable reliability, cause of high individual errors [58]
		Errors of joint repositioning [59]
Electro-goniometer (angle)	High reliability [57]	Large non-linearity and hysteresis [57]
Accelerometer (linear acceleration)	Stable and easy to attach. high sensitivity and excellent reliability of the pendulum test [43] less expensive; not restricting the movement [57]	Less accurate angle estimation during movement [59]
Gyro (angular velocity)	No need for numerical differentiation; sufficient accuracy; low susceptibility to effects from the motion of the knee joint axis; no restriction of the knee joint when worn; simple and stable attached; and ability to obtain waveforms of angle, angular velocity, and angular acceleration simply and with high accuracy [57]	Stability and reliability remain problematic [57]
Inertial Motion Units (IMUs, acceleration plus angular velocity)	Simple use	Issues concerning the validity and reliability of the measurements
Camera-based methods	Simple use	Difficult video analysis

### Outcome measures of pendulum test

Alongside development of technologies of implementation for pendulum test, numerous measures were suggested to classify the severity of spasticity as outcome measures. Wartenberg used the test qualitatively paying attention to irregularities in swinging time or the number of oscillations. He also suggested Resting position as his method’s outcome measure. After that, many measures were introduced by each research group. A main goal of this review was to standardize the names and definitions that are given to the same aspect of pendulum-like oscillation (outcome measures) which does not look the same and is a source of confusion. This was done by grouping the all outcome measures based on the collected signals which are angle, angular velocity, and angular acceleration. Then, every possible category of suggested outcome measure, with all different names and definitions are brought under that group. This was performed in Table 5.

**Table 5:** Main categories of outcome measures (parameters) that are suggested in the studies that used pendulum test

Signal	Category #	Parameter	Measure/parameter definition	Refs.
Angle	1	Onset Ang	Angle at the start of test response	[19]
		A0	Knee angle at the beginning of the test during maximal limb extension	[13, 35, 47]
		αs initial angle	Initial Knee angle	[41]
	2	F1 Ang	Angle at the end of initial movement into flexion	[19, 36]
		F1 Amp	F1 Ang—Onset Ang	[19, 36]
		Ex	First swing excursion: the difference between the starting angle (the position at which the examiner released the participant’s heel) and the first angle of reversal of the swinging limb	[59]
		P4	First maximum of the oscillation	[38, 45]
		FAR	knee angle when shank motion first switched from flexion to extension	[18]
		A1	The amplitude of the first swing	[38, 44]
			Magnitude of first drop	
		ϕmax	The first maximum of the goniogram after releasing the leg	[35]
		αp	The peak angle of the first swing	[41]
		Θ1 first reversal	First maximum of the oscillation	[6, 46]
	3	E1 Ang	Angle at the end of initial movement into extension	[38]
		E1 Amp	F1 Ang—E1 Ang	[38]
		A2	The angular change between the first minimum and second maximum	[35, 40]
	4	Rest Ang	Resting knee angle	[6, 18, 19, 44, 46]
		Rest angle (RA) *θr*	Knee angle at the end of oscillations	
		Plat Amp	Rest Ang—onset Ang	[19, 36]
		αf	Final position of the leg	[41]
	5	Duration	Duration of oscillations	[6, 43]
		T	Test duration	
		Relative Swing Time	The time between the peaks, Normalized to the height of the person	[17, 29]
		TFR	Time to first reversal: time interval between the start of shank motion and the first reversal from flexion to extension	[18]
		Test Duration	Duration from onset Angle to rest angle	[19]
	6	F	Frequency of oscillations	[35, 44]
			Frequency of the swing	
		C1 Freq	Initial cycle frequency = 1/duration of E1 Ang	[19]
		N	The number of sinusoidal waves produced by the swinging limb after the heel was released (minimum of 3 degrees)	[6, 37, 38, 45, 56]
		P2	The Number of swings	
		Ncyc	Number of cycles (full oscillations) was counted between start of motion and until the oscillation amplitudes is less than 3 degrees	[46]
	7	RI	Relaxation index: (starting angle − first angle) / (starting angle − resting angle)	[28, 45, 56]
			F1 Amp / Plat Amp	[19]
			Θ1 / *θr*	[6]
		ERI	Extension Relaxation index = E1 Amp/ Plat Amp	[19, 36]
		*β*	Damping ratio. Defined as the ratio of the logarithmic decrement (*δ*) to the period; expressed in sec	[43]
		*λ*	Defined as the natural log of the second to fourth peak amplitude ratio	[43]
		P1	Normalized relaxation index	[38, 45]
		P5	Relaxation index at the half swing	[38, 45]
		P6	Average relaxation index of 10 successive swings	[38]
		Ratio 1	Ratio 1: A1/(A1 − A2), where A1 is the amplitude of the first oscillation and A2 is the amplitude of the second oscillation	[18, 28, 38, 52, 58]
		Ratio R1	Ratio R1: A/B, where A is the amplitude of the first oscillation and B is the amplitude of the second oscillation	
		R1 ratio	The amplitude of the first swing (A) divided by the amplitude of the rebound angle	
		Ratio 2 Ratio R2 R2 ratio	Ratio 2: A1/A0, where A0 is the final resting angle and A1 is the amplitude of the first oscillation	[17, 29, 34, 38, 40, 42]
			R2: first swing (A) divided by the amplitude of the final position (C)	
		R2n	The normalized relaxation index. R2n: A1/1.6A0, where A0 is the knee angle between the full extension (starting position) and the neutral knee joint angle (end position), and A1 is the difference between the starting angle and the maximum flexion	[35, 38, 41]
	8	AUC	Area Under Curve: area between the knee angle during oscillations and the resting angle; it is the integral of the absolute value of the knee angle	[18, 38, 46]
		P3		
		Ptotal	The area between the goniogram and the time axis	[35]
		Relative area difference	Relative difference |P + − P-|/Ptotal between the positive and negative areas and the total area between the goniogram and the neutral line starting from the first minimum	[35]
	9	PT	Total pendulum Score (combination of multiple parameters)	[35]
Angular velocity	1	*ω*max	The maximum angular velocity of the shank	[35]
		*V*max	maximal velocity of the first swing (°/s)	[17, 28, 29]
		F1 Vel	Maximum velocity during F1 Amp	[19]
		P7	First maximum of the tachogram	[38]
	2	VFR	Velocity to first reversal: VFR = FAR / TFR	[18, 46]
	3	E1 Vel	Maximum velocity during E1 Amp	[19]
		P8	First minimum the tachogram	[38]
		ωmin	The minimum angular velocity of the shank	[35]
Angular acceleration	1	F1 A/D ratio	Initial flexion acceleration/deceleration ratio = Max acceleration duration F1 Amp/ Max deceleration during F1 Amp	[19]
		First Maximum Acceleration	First Maximum Acceleration during flexion phase	[28]
		Max Acc on the rebound swing	Max Acceleration during extension phase	[28]

The first mentioned set of outcome measures from pendulum test of spasticity on the knee were by Boczko et al. [27] in 1950s. During the following half a century, other than number of oscillations, test duration and peak angles, measures such as relaxation index (RI) were introduced to have higher reliability. The most comprehensive works on outcome measures during this time were performed by Bajd et al. [37–39] and Stillman et al. [19]. Table 5 illustrates all categories of outcome measures that have been suggested in the literature of pendulum test of spasticity. The three main categories are parameters based on angle data, based on angular velocity, and finally based on angular acceleration. All the investigated studies have used outcome measures that are based on joint angle during the test. Almost half of the studies have used parameters that are based on angular data (Table 5). A considerable amount of the studies has used more than one category (Table 5).

Overall, the suggested parameters can be divided into two categories of primary and secondary measures. Primary measures are directly obtained from aspects of the collected data such as the peak value of an angle, or number of oscillations (for example the first six categories in Table 5 for parameters based on angle data). Secondary outcome measures are calculated or combined from the primary ones mainly to reduce the number of parameters and to come up with ones that are more sensitive or better illustrate level of spasticity.

Outcome measures that are based on angle data are divided into 9 categories in Table 5. Category #1 is based on the initial position of the extended knee in the test. Although 29% of studies (out of 17 that we have investigated) have used this category in their set of parameters, it is not an outcome measure of spasticity test by itself, but is mainly used in the calculation of other outcome measures. Second category is used by 71% of the studies and relies on the maximum flexion angle in the first swing after the leg release. Category #3 examines the max angle in the first rebound towards extension of the knee, which is used by 18% of the studies. Resting angle or the final position of the leg is category #4 and is used by 41% of the literature. Category #5 examines durations in the test, whether overall duration or the time between two specific points, and is used by 35% of the studies. Number of cycles or frequency is category #6 and is used by 59% investigators. In counting the oscillations, a threshold of three degrees is considered below which the oscillations are ignored. Frequency concept was either used as the inverse of the time duration for the first cycle, or as the ratio of the number of total cycles to the total time. Time and frequency categories could be based on angular velocity or acceleration during the test, but almost all the researchers have based them on the angle data. Categories #7–9 are secondary measures and are calculated from the previous categories. Category #7 examines the ratio of the amplitudes for various angles. Wide range of ratios have been suggested by researchers and almost all studies (94%) have used at least one such category of outcome measure. Most of the powerful and consistent outcome measure that were capable of identifying levels of spasticity were in this category [40, 41]. Category #8 uses area under the knee angle curve which considers positive and negative values for flexion and extension around the resting angle, and is used by 24% of the studies. Recently, Popovic et al. suggested a total score for pendulum test which takes many aspects of the angle cure into account and comes up with a final score [35]. This is category #9 in Table 5.

There are three categories of outcome measures in the angular velocity group. Category #1 uses the first maximum angular velocity in the flexion direction, and is used by 35% of the investigators. Category #2 relies on the average angular velocity in the first swing towards the flexion, and is used by 12% of the literature. Category #3 is based on the first maximum velocity in the rebound towards the knee extension, and is suggested by the 18% of the literature. Some of the researchers call this category the minimum velocity instead of maximum velocity in the opposite direction.

Few studies have used outcome metrics that rely on angular acceleration. Stillman et al. [19] suggested the ratio of maximum acceleration to maximum deceleration during the first swing towards flexion of the knee as their measure of spasticity. Maximum angular acceleration both in flexion and in extension phases are separately used as outcome measures by Brown et al. [28] in 1988.

As can be seen from the table, quite a lot of parameters have been suggested as outcome measures. However, it is not practical to use all of the suggested parameters, and unfortunately no study has compared all these parameters to provide a comprehensive subjective scale of strength/weakness points. Although in Table 5 we provided an exhaustive list of outcome measures, in separated categories of measure signals, in Table 6 we introduce list of key publications that have used more than one outcome measure. In this way, pioneers in using popular sets of outcome measures are highlighted. Table 6 is the list of the main studies on the sets of outcome measures of pendulum test that is used in this review.

**Table 6:** Sets of outcome measures that were proposed in each key study

# of used parameters	List of parameters (measures)	Author/year
8	p1–p8 (using A0, A1, A2, R1,R2,R2n)	Bajd, 1984 [38]
6	Ex, RI, *β*, *λ*, *t*, *n*	Szopa, 2014 [43]
4	p1, p2, p4, p5	Yeh, 2016 [45]
1	R2n index (using αf, αs, αp)	Vargas Luna, 2016 [41]
4	RI, Test duration, Fang, Rest ang	Azevedo, 2013 [55]
2	Ratio 1 & Ratio 2	Bui, 2017 [40]
1	PT score	Popovic-Maneski, 2018 [35]
2	Ratio R1, Ratio R2	Leslie1992 [42]
5	Θ1, N, Duration, RI, θr	Greenan Fowler, 2000 [6]
4	R2 ratio, R1 ratio, Vmax, Relative swing time	Nordmark, 2002 [17]
4	First Reversal, AUC, VFR, Resting Angle	Bohannon, 2009 [46]
5	FAR, IA, AUC, TFR, VFR	Sterpi, 2013 [18]
14	On Ang, Rest Ang, F1 Ang, E1 Ang, F1 Amp, E1 Amp, plat Amp, RI, ERI, F1 Vel, E1 Vel, F1 A/D ratio, Duration, C1 Freq	Stillman, 1995 [19]
5	F1 Amp, E1 Amp, plat Amp, RI, ERI	Tancredo, 2013 [31]
4	RI, Vmax, First Maximum Acceleration, Max Acc on the rebound swing	Brown, 1988 [28]
3	A0, A1, Resting Angle	He, 1997 [44]
6	RI, ERI, F1Amp, E1Amp, Plat, Ncyc	Whelan, 2018 [36]

**Fig. 3:**
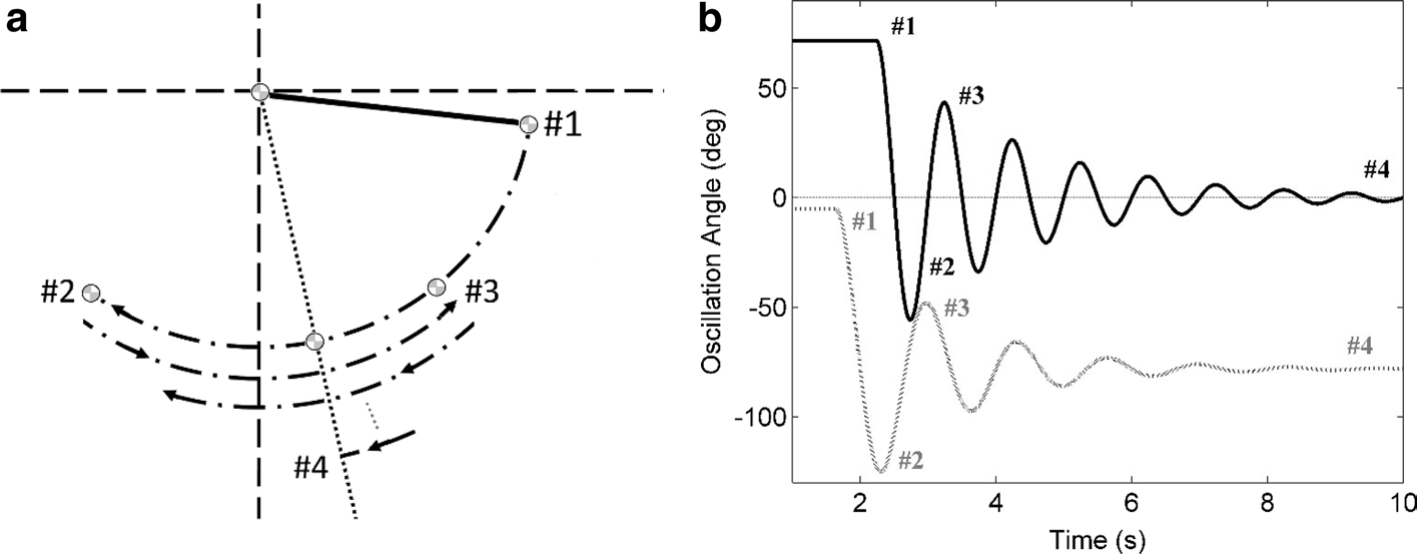
**a** First couple of swing traces along the first four category of outcome measures (#1 to #4) that are key joint angles during the pendulum test and explained in Table 5. **b** Time series for the knee joint angle during the pendulum test along the first four category of outcome measures. The bold trace corresponds to when the reference is the resting angle. The dotted trace corresponds to when the reference is horizontal plane

In the literature of the pendulum test for spasticity, there is no standard agreement between the researchers particularly in definition of the outcome measures. One main reason for this ambiguity is the different references used for measuring the knee angle. Overall, four key positions can be used as a reference: Horizontal plane, vertical plane, initial shank position, and final shank position in the test. Figure 3 shows the difference in the collected data with different experimental methodologies. Figure 3a shows these four possible frames of reference for knee angle, along the first four categories of angle parameters in Table 5.

Most of the studies have used horizontal plane as their frame of reference [6, 17–19, 28, 31, 42, 43]. The second most popular frame of reference for angular movement is rest angle or the final position of the knee in the pendulum test [29, 34, 35, 38, 44, 45]. Some of the researchers have used the initial angle of the shank as their reference instead of horizontal plane [36, 40, 46]. No researcher has used vertical plane as their reference in knee angle measurement. One example of such ambiguity is A0 which depending on the reference is defined as “Final resting angle” [40] as well as “knee angle at the beginning of the test during maximal limb extension” [18, 44]. Similarly, differences can be observed in the definition of other spasticity measures such is definition of RI in [6, 19] and in [38, 45].

Figure 3b illustrates two typical knee angle traces during a pendulum test. The bold trace corresponds to when the reference is the resting angle. Sections of knee angle trace, that parameters (angle outcome measures) in categories #1 to #4 are extracted, are marked. The dotted trace corresponds to when the reference is horizontal plane. If the beginning of trace falls on the time axis, then the reference is initial angle.

## Conclusions

This systematic review aimed at analyzing the state of the art in implementation of Pendulum test and the outcome measures of this method of evaluation of spasticity. This test is agreed to be repeatable and a valid method of identifying presence of spasticity [29, 46], though it may not be successful in discriminating between close levels of spasticity [36]. Since lower leg weight is suitable to create enough speed through free fall in the pendulum test, which is crucial for this speed dependent symptom, most of this test’s applications is on the knee joint. However, using extra weight and special apparatus, this test can be applied to other joints such as elbow [47].

Non-instrumented pendulum test lacks objectivity and is apparently not used much by researchers. Instrumented pendulum test using multitudes of sensor technologies have been reported in the literature and evaluated as a feasible method of objective assessment of spasticity particularly for the knee extensors [19, 48, 49].

Sensor technologies that are used for implementation of this test (Tables 3, 4) would affect reliability/repeatability, ease and speed of setup, size and hindrance to pendular movement and hence suitability for clinical use, susceptibility to interfering factors such as gravity, ease of data processing and analysis and hence possibility of implementation in stand-alone devices. Overall, in the past few decades, the most technology been used is goniometer and electro-goniometer. Choice of sensor affects the type of signal that is used in data collection and thereby in outcome measure for the test. Although, processing techniques such as differentiation can transform angle data to angular velocity or acceleration. As can be seen in Table 5, all of the studies that we found to provide objective assessment method of spasticity with outcome metrics, used angular data. Almost half of them have also used angular velocity whether using tachometer/gyroscopes or differentiating angular data. Finally, almost 10% of the studies have focused on angular acceleration data as well.

We have categorized all 33 outcome measures that appeared in the literature into 13 categories in Table 5. These measures/metrics are supposed to help pendulum test differentiate between healthy and various levels of spasticity in the joint under consideration. A recent study also showed that the history of movement also affects the results of pendulum test [50]. Some of the studies have commented on the sensitivity of single outcome measures [6, 36, 51]. Each of the studies we investigated utilized between one to 14 outcome measures (Table 6).

Most of the studies have used more than one measure and argue that no single measure can represent all abnormal aspects of passive resistance of the disordered joint to the movement [19]. Unfortunately, there has been no study that optimizes a model using all or some of these 33 measures and comes up with a single combined measure with the highest discriminative power. Although such a model might be different for various spastic groups of patients. Nonetheless, some of the studies have tried such modeling approach without doing the optimization step. For example, Whelan et al. tried two models to find presence of knee spasticity in four different patient cohorts [36]. Another example is the attempt by popovic-Maneski et al. [35] in providing a single measure called global measure of spasticity.

Finally, such metrics that come out of pendulum test is supposed to be used by clinicians and should prove its power in a comparison by their golden clinical measures. Out of 5 clinical scales that are mentioned in studies we investigated, MAS and AS were the mostly used ones (Table 3). However, a modeling study that provides a relationship between such clinical scale as a gold standard and a single/combined outcome measure is really missing.

In this review we tried to come up with a comprehensive list of outcome measures along with the suggested sets of measures by lead researchers in this field (Tables 5, 6). We tried to provide standardized definitions for outcome measures through 13 categories for three type of signals (Table 5). However, it seems that a thorough modeling study is still missing. One that tries to take all of the 33 outcome measures into account and provides the best minimal sets of necessary parameters. Such final model, if provided, will accelerate making acceptable clinical device for objective assessment of spasticity. It goes without saying that there should be a simulation study on a mechanical pendulum with adjustable parameters, or alternatively a clinical experiment on patients with the whole range of spasticity before such optimization study can be performed.

## Methods

### Search strategy

A systematic review was conducted to identify literature published in four international databases between 1950 and the end of 2019: PubMed, Science Direct, Google Scholar and IEEE explore. Science Direct database was available from 1995 and hence was searched from 1995 to 2019. Combining the keywords “pendulum test” and “Spasticity” is defined as the search strategy. No filters were applied on the type of disease, because it was not the focus of study. The search strategy was the same in all databases, with modifications to fit the web interfaces. In other words, writing the queries or combination of Boolean operators could be modestly different for each database. Figure 1 shows the search strategy and selection process.

### Study selection and eligibility criteria

After excluding duplicates, the selection of studies was carried out in two stages. All the remained articles were screened first by title and abstract, and then by full text for eligibility. At least one of the mentioned sections should include both “pendulum test” and “spasticity”, and also there should be a mention of quantitative/objective assessment, or instrument/sensor, or outcome measure. In other words, pure clinical studies with no quantitative evaluation of spasticity were excluded.

## Data Availability

The generated or analyzed data during this study are all included in the published article.

## Abbreviations

CSI: Composite Spasticity Index
HAT: Hypertonia Assessment Tool
TSS: Triple Spasticity Scale
AS: Ashworth Scale
MAS: Modified Ashworth Scale
TS: Tardieu Scale
MTS: Modified Tardieu Scale
ROM: Range Of Motion
MS: Multiple Sclerosis
CP: Cerebral Palsy
SCI: Spinal Cord Injury
RI: Relaxation Index
NC: No Comparison
DS: Degenerative Syndromes of the nervous system
NR: Not report in original paper
PTD: Pendular Test Device
DAROM: Dynamic Evaluation of Range of Motion scale
AIS: Association Impairment Scale
ARSACS: Autosomal Recessive Spastic Ataxia of Charlevoix/Saguenay
IMU: Inertial measurement Units
ABI: Acquired Brain Injury
SDR: Spastic Diplegia undergoing selective dorsal Rhizotomy
Ang: Angle
AMP: Amplitude
Ex: Excursion
TFR: Time of first reversal
F, Freq: Frequency
N: Number of oscillation
Ncycle: Number of cycle
ERI: Extension Relaxation Index
AUC: Area Under Curve
Vel: Velocity
PT: Total Pendulum Score
RA: Rest Angle
EMG: Electromyography
CNS: Central Nervous System
